# Modulation of Spectral Representation and Connectivity Patterns in Response to Visual Narrative in the Human Brain

**DOI:** 10.3389/fnhum.2022.886938

**Published:** 2022-10-06

**Authors:** Zahraa Sabra, Ali Alawieh, Leonardo Bonilha, Thomas Naselaris, Nicholas AuYong

**Affiliations:** ^1^Department of Neurosurgery, Emory University, Atlanta, GA, United States; ^2^Department of Neurology, Medical University of South Carolina, Charleston, SC, United States; ^3^Department of Neuroscience, University of Minnesota, Minneapolis, MN, United States

**Keywords:** visual narrative, brain connectivity, Spectral representation, SEEG, contextual novelty

## Abstract

The regional brain networks and the underlying neurophysiological mechanisms subserving the cognition of visual narrative in humans have largely been studied with non-invasive brain recording. In this study, we specifically investigated how regional and cross-regional cortical activities support visual narrative interpretation using intracranial stereotactic electroencephalograms recordings from thirteen human subjects (6 females, and 7 males). Widely distributed recording sites across the brain were sampled while subjects were explicitly instructed to observe images from fables presented in “sequential” order, and a set of images drawn from multiple fables presented in “scrambled” order. Broadband activity mainly within the frontal and temporal lobes were found to encode if a presented image is part of a visual narrative (sequential) or random image set (scrambled). Moreover, the temporal lobe exhibits strong activation in response to visual narratives while the frontal lobe is more engaged when contextually novel stimuli are presented. We also investigated the dynamics of interregional interactions between visual narratives and contextually novel series of images. Interestingly, the interregional connectivity is also altered between sequential and scrambled sequences. Together, these results suggest that both changes in regional neuronal activity and cross-regional interactions subserve visual narrative and contextual novelty processing.

## Highlights

Our results demonstrate that while multiple cortical regions including the frontal, parietal and temporal lobe respond to visual narrative stimuli, they do so differently. Our main significant finding is that in the human brain, there is a concerted but inverse response at the frontal and temporal lobe to semantic relationship and contextual novelty. While the temporal lobe exhibits strong activation in response to visual narratives, the frontal lobe is more engaged when stimuli is contextually novel. The interregional connectivity between these cortical regions is also modulated between these two stimuli conditions. Consequently, these findings are relevant in further understanding neurological conditions where visual semantic processing or contextual novelty detection is impaired.

## Introduction

Pictorial storytelling is a critical aspect of human cultural development, spanning from the earliest prehistoric cave art examples (Callaway, 2019) to the “visual narratives” widely seen in today’s advertisements and digital animations. Visual narratives consist of a sequence of static visual images linked semantically through event-related chronology, contextual consistency, and content interaction across scenes (Cohn, 2014; Loschky et al., 2020). As human beings, we can readily recognize visual narratives as being inherently different from a set of random images. Yet, this seemingly effortless cognitive distinction requires the complex integration of multiple neural processes for object detection, attentional selection, and memory.

The cognitive processing of visual narratives involves detecting “contextual novelty” where an image with familiar features is encountered in an unexpected manner (Ranganath and Rainer, 2003; Schomaker and Meeter, 2015). Contextual novelty detection requires awareness of both visual and semantic serial dependencies among previously seen images (Huffman et al., 2017) within a time-window associated with the narrative (Worthen, 2006; Kiyonaga et al., 2017). Some studies have used intracranial electrocorticography (ECoG) to study novel object recognition to clarify the roles of selected brain regions. For instance, using movies as stimuli, neural activity in the occipital and temporal lobes have been shown to encode whether a movie frame belongs to the same movie, as early as 100–200 ms after presentation (Isik et al., 2018). Investigation of novel object detection using ECoG recordings from human subjects (Miller et al., 2015) showed that visual category-selective subregions within the ventral temporal lobe, including face-selective fusiform gyrus loci and place-selective parahippocampal/lingual gyrus loci, exhibited differential response to contextual novelty. Novel stimuli resulted in increased broadband spectral power, which indexes the underlying population spiking activity (Manning et al., 2009; Whittingstall and Logothetis, 2009; Miller, 2010; Crone et al., 2011), in both object category-selective sites. These prior studies have predominantly focused on investigating a specific brain region, mainly temporal or occipital lobes.

While novelty processing has been implicated in specific brain regions (Rolls et al., 2005; Kishiyama et al., 2009; Kumaran and Maguire, 2009; Axmacher et al., 2010; Davachi and DuBrow, 2015; Isik et al., 2018), the coordinated interactions among several interconnected brain regions/networks are thought to be involved in novel visual information processing (Knight and Nakada, 1998; Ranganath and Rainer, 2003; Kafkas and Montaldi, 2018). Prior work in humans and primates have implicated a spatially distributed network of brain regions including both the anterior and posterior multimodal association cortices in detecting novel stimuli or contextual novelty. The frontal lobe is well known to be heavily involved in detecting novel, mismatched or unexpected stimuli (Courchesne et al., 1975; Rolls et al., 2005; Kishiyama et al., 2009; Cohn and Kutas, 2017). Medial temporal lobe structures also exhibit differential engagement when processing novelty and contextual information (Axmacher et al., 2010; Kafkas and Montaldi, 2018; Fonken et al., 2020). Also involved are the brain areas comprising the ventral visual stream, spanning from the occipital lobe to the temporal lobe (Kafkas and Montaldi, 2018). As with visual receptive fields increasing in size and feature complexity along the visual system hierarchy (Hubel and Wiesel, 1962; Desimone et al., 1984; Goodale and Milner, 1992; Grill-Spector and Malach, 2004), fMRI and electrophysiological studies in human subjects (Hasson et al., 2008; Lerner et al., 2011; Himberger et al., 2018) have similarly found that higher brain regions along the visual hierarchy employ a wider temporal window to integrate visual information, termed temporal receptive window (TRW; Yeshurun et al., 2021). Whereas cortical neuronal circuits involved in the early stages of sensory processing are both sensitive and responsive to the fast-changing low-level stimuli features, the TRW widens along the sensory processing pathway where higher-level cortical areas are increasingly able to integrate information accumulated over a much longer timespan. The reliability of human neuronal population responses along the visual cortical hierarchy for tracking shifting visual stimuli information at different timescales was examined by Honey et al. using ECoG signals in human subjects while watching intact and scrambled movies (Honey et al., 2012). They demonstrated that in early visual areas, high-frequency gamma power (64–200 Hz) power was informative about movie stimuli type (intact vs. scrambled) whereas in higher order regions, power fluctuations were more reliable for unscrambled rather than scrambled movies. The processing of scrambled and intact auditory stimuli (Lerner et al., 2011; Davidesco et al., 2018) similarly involves population neuronal operations at differing time-scales along the sensory processing hierarchy and memory system (Hasson et al., 2015).

Theoretical models are also useful frameworks for studying how distinct neural processes jointly participate in the cognition of visual narrative (Cohn, 2020c; Loschky et al., 2020). The Parallel Interfacing Narrative-Semantics (PINS) model proposed by Cohn (2020c) describes concurrent narrative and semantic representational levels for processing visual narratives. Semantic processing involves object recognition and selection of salient features in images. Narrative processing involves organizing seen images according to context in working memory. Seminal work by Cohn (2014); Cohn and Kutas (2017) using surface electroencephalograms (EEG) recordings have described three features of event-related potentials (ERPs) that are associated with components of the visual narrative based on the PINS model (Cohn, 2020c). These include the N400 peak in the midline central leads elicited by semantic incongruency, anterior negativity in the midline prefrontal leads elicited by narrative structure, and a P600 peak in the midline parietal leads that is sensitive to concurrent narrative and semantic processing (Cohn et al., 2012; Cohn and Kutas, 2017; Cohn, 2020c). Loschky et al. proposed a model based on the Scene Perception & Event Comprehension Theory (SPECT) with iterative crosstalk between front-end and back-end processes. The front-end processes involve information extraction from seen images and attentional selection of salient features within the visual story. Back-end processing, occurring simultaneously, uses input from the front-end processes as well as semantic memories to develop a cognitive model and updates this model as new information arrives (Loschky et al., 2020).

Common to both models are the multifaceted processing mechanisms for concurrent comprehension of the visual narrative and detection of contextual novelty. As novelty processing has been implicated in several brain regions (Rolls et al., 2005; Kishiyama et al., 2009; Kumaran and Maguire, 2009; Axmacher et al., 2010; Davachi and DuBrow, 2015; Isik et al., 2018), it remains fuzzy if cognition of visual narrative in the human brain also engages nodes that constitutes the novelty-detection neural network. This work will specifically investigate how regional cortical activity and cross-regional interactions between the frontal and temporal lobes support visual narrative interpretation using intracranial stereotactic EEG (sEEG) recordings from human subjects. Our task requires study subjects to distinguish between “sequential” images presented from the same story/fable or “scrambled” images drawn from multiple fables. The manipulation of sequential vs. scrambled visual sequences has been employed in previous work to study the comprehension of visual narrative (Gernsbacher et al., 1990; Robertson, 2000; Cohn et al., 2012) and movie stimuli (Honey et al., 2012). We specifically examined if regional neural activity exhibits spectral features specific to processing of sequential vs. scrambled image patterns and whether interregional connectivity changes in response to scrambled or sequential images. Our results demonstrate that broadband spectral activity spanning sub-gamma (<30 Hz) along with gamma frequency range (Miller et al., 2014; Sabra et al., 2020), mainly within the temporal and frontal lobes, encodes whether presented images are part of a visual narrative or random set with high accuracy. The temporal lobe is highly engaged when the stimuli have predictable semantic structure while the frontal lobe exhibits stronger activation in response to novelty. Furthermore, the dynamics of interregional connectivity is also modulated between sequential and scrambled sequences suggesting that alterations to both regional neural activity and cross-regional interactions between the frontal and temporal lobes subserve visual narrative processing.

## Materials and Methods

### Participants

Thirteen patients with epilepsy (7 males, 6 females) with ages between 14 and 66 years old (mean age 35) participated in these experiments. All subjects underwent surgical implantation of intracranial sEEG electrodes for clinically indicated invasive neurodiagnostic for evaluation of seizure source. One subject (S2) also underwent subdural grid electrode implantation over the lateral surface of the right hemisphere in addition to depth electrodes. The location of the implanted electrodes was determined by both epilepsy neurologists and neurosurgeons, based solely on clinical considerations. All subjects provided written informed consent as approved by the Institutional Review Board at the Medical University of South Carolina.

For the duration of the experiments, subjects sat in an upright position and viewed a laptop screen placed at eye-level at an approximate distance of 40 cm. Subjects were able to take breaks and can stop participating at any time during the experiment.

### Recording Sites Specifications and Locations

Intracranial electrode locations differed between subjects according to their respective clinical indications (Supplementary Table 1). Each depth electrode (Ad-Tech, Oak Creek, WI) has 10 recording contacts, with a diameter of 2.29 mm and a 5 mm distance between adjacent recording contacts. All participants underwent depth electrode placement only except for participant S2 who had an additional subdural grid placed also. (Ad-Tech, Oak Creek, WI, United States; 6 × 6 recording contacts with 10 mm spacing between contacts).

Post-implantation T1-weighted structural magnetic resonance (MR) images were used to determine the anatomical location of each electrode’s recording site. We masked the electrodes in the structural images using “cost function masking” (Brett et al., 2001) in MRIcron (MRIcron, RRID:SCR_002403). The Clinical Toolbox (Rorden et al., 2012) within SPM8 (SPM, RRID:SCR_007037; Clinical Toolbox for SPM, RRID:SCR_014096) was used to normalize the masked structural images of each subject into Montreal Neurological Institute (MNI) space. The recording sites in MNI space were visualized using BrainNet Viewer (BrainNet Viewer, RRID:SCR_009446) with surface template ICBM152. Recording sites reported to be source of seizures were excluded from the analysis (refer to Supplementary Table 1 for epileptic zones), and the anatomical locations of the remaining 970 recording sites across all subjects were distributed as follows: frontal (ten subjects, 176 in left hemisphere, 179 in right hemisphere), temporal lobe including medial temporal lobe (thirteen subjects, 201 in left hemisphere, 195 in right hemisphere), insula (seven subjects, 15 in left hemisphere, 28 in right hemisphere), occipital lobe (two subjects, 8 in left hemisphere, 15 in right hemisphere), parietal lobe (twelve subjects, 41 in left hemisphere, 78 in right hemisphere), and basal ganglia (seven subjects, 21 in left hemisphere, 13 in right hemisphere).

### Data Recording

Local field potential (LFP) recordings were recorded using a clinical XLTEK EEG system (Natus Medical, Inc.). For all subjects, the sampling frequency was 2 KHz except for subject S2 where it was 500 Hz. Data recorded at 2 KHz sampling rate were resampled to 1 KHz for offline analysis.

To synchronize the onset of the image stimulus on the screen with the intracranial recordings, a photodiode was used to detect color changes within a small area on the laptop screen at the onset of each new stimulus. The photodiode was placed at the bottom right corner of the screen and was obscured to avoid distracting the subjects during the experiment. Each presented image stimulus is transduced by the photodiode as a voltage pulse that is recorded along with the intracranial signals. The time series of triggered pulses from stimuli onsets of an experiment were used to perform offline synchronization between the timing of image onset on the screen and the corresponding neuronal activity recorded across the electrodes’ contacts (Rorden and Hanayik, 2014).

### Design of Visual Narrative Experiment

**FIGURE 1:**
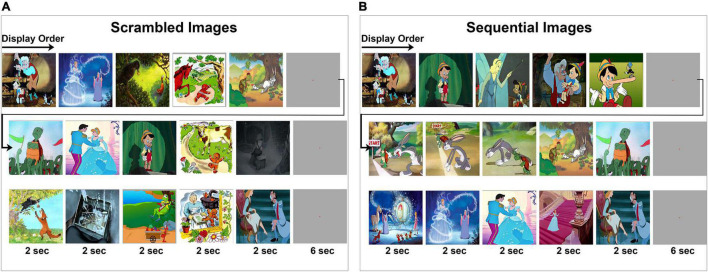
Visual experiment design. Subjects viewed a series of cartoon images that are part of five fables under two experimental conditions. Displayed is a subset of the image sequences. Within each row, the chronological order of image presentation is from left to right as indicated by the arrow. **(A,B)** A total of seven image series were presented where each series consisted of five images presented for 2 s each, followed by a gray screen for 6 s duration before the start of the next image series. The full set of images are provided in Supplementary Figures 1, 2. **(A)** Subjects were first instructed to fixate at the center of the screen while a series of images are presented in scrambled order. **(B)** Then, subjects were instructed to fixate at the center of the screen while the same images are presented in a sequential order.

A total of 35 cartoon images belonging to seven different fables were used in these experiments. Each fable was represented by five images. Subjects were given instructions to view images as they were presented on the laptop screen. An experiment has two parts consisting of a “scrambled” and a “sequential” experimental condition (Figure 1). Subjects were explicitly instructed to view the images as they appear. In the first part of the experiment (Figure 1A), images were presented to the subjects in “scrambled” order where images from multiple fables were intermixed and displayed in sets of five images. In the second part of the experiment (Figure 1B), the same images that were presented during the scrambled phase were presented in their “sequential” or correct chronological order according to each fable’s narrative context. Each set of five images was shown in sequential order corresponding to the semantic and structural coherence of the original story plot of events (Cohn, 2020c; Loschky et al., 2020). During both parts of the experiment, each image was displayed for 2 s. A gray screen of 6 s duration was used to separate each set of five images. In each part of the experiment (scrambled and sequential), a total of 35 images were displayed one time without repetition. Analysis was carried out in a time window inclusive of the transition between two consecutive images, spanning from 100 ms during the previous image, before the onset of the next image, to 500 ms following image transition. This analysis window was chosen to study the LFP associated with the transition between two images rather than the processing of fixed image stimuli per se. Block design was used in this experiment since it is easier and less challenging for the participating subjects to understand. All 13 subjects were debriefed at the end of the experiment and they all confirmed familiarity with the presented fables and confirmed the ability to notice that the images in the first and second parts of the experiment were presented, respectively, in a scrambled and sequential order.

### Experimental Design and Statistical Analysis

#### Signal Preparation

Recording sites identified by the patient’s neurologists to have ictal or high interictal epileptiform activity were excluded from the analysis. Additionally, we also excluded recording sites found on post-implantation structural MR images to be outside of the brain’s cortical surface and also those whose anatomical location is not clearly identifiable. Based on these criteria, 300 recording sites were excluded while analysis was carried out on 970 recording sites across subjects.

For each subject, each electrode signal was re-referenced to the global mean signals over all included electrodes by subtracting the average amplitude at each time point from the individual recorded time series signal at each site.

#### Power Spectra Calculation

Trial-based analysis for each recording site was carried out using 600 ms time windows, starting 100 ms before each image stimulus onset to 500 ms following the image transition. Analysis was confined to the resulting single trial-power spectral density (PSD) in the frequency range from 2 to 100 Hz using the PWelch method (Welch, 1967) at 1 Hz increments. The default Hamming window in MATLAB (Mathworks, MA, United States) was used to obtain eight segments of the time series data, with 50% overlap between the segments. Frequencies between 56 and 63 Hz were excluded to eliminate 60 Hz line-noise artifacts. For each recording site, the calculated PSD was normalized to the average power spectra across all experimental conditions for that recording site (i.e., taking the power values of the trial-PSD at each frequency and dividing it by the average power value at that frequency across all experimental conditions). Trial-PSDs were separated into two groups based on the experimental condition (i.e., scrambled or sequential) Average power spectra for each experimental condition were calculated for each recording site for each subject and across subjects, resulting in 1940 PSDs derived from 970 recording sites across all 13 subjects. All the analysis was carried out over a 2–100 Hz frequency range, as the usage of a wider range (2–200 Hz) did not reveal any difference in the results (data not shown).

#### Dimensionality Reduction

Dimensionality reduction was performed using principal component analysis (PCA; Pearson, 1901). Principal components (PC) were calculated for the 1940 PSDs. The first and the second principal component accounted for 76% and 7% variance respectively across the PSDs.

#### Principal Components-Projection Encoding Model

The spectral features of recorded neural activity related to the observation of scrambled (Scr) and sequential (Seq) images was further explored using a PC-projection encoding model. Analysis was carried out separately for each recording site and each subject. For a given subject *s*, we applied PCA to all PSDs derived from the other 12 subjects and calculated the projection onto the resultant first principal component (PC1) for each single-trial PSD of subject *s*. Since we are interested in the response during the transition between two consecutive images, analysis of each recording site’s response was examined during transition between two sequential images of the same visual narrative vs. two scrambled images from different stories with no expected narrative context. Responses to the first presented image of the experiment and to the gray screen (Figure 1) were omitted. For each recording site, the accuracy of spectral features for encoding Seq vs. Scr transitions was tested using an encoding model derived from PC1 projections. Encoding models were generated using 80% of the trials to train and validate using Monte Carlo cross-validation (Dubitzky et al., 2007; Kuhn and Johnson, 2013). The remaining 20% of the trials were used as a test data set to assess the decoding performance of the derived model. The Pearson’s Correlation between predicted projections derived from the training set and measured projections in the validation set was calculated to assess encoding accuracy. Formally, for a given recording site, let **q**_*i*_ be the *i*th single-trial PSD, and **p** the PC1 component. Thus **y**_*i*_ = **q**_*i*_⋅**p** corresponds to the projection of the *i*th single-trial PSD onto PC1. A linear regression was then applied using the training projection values to calculate the predictive weights of the two experimental conditions Scr and Se:


y=X⁢β+ε

where y is the vector of projections (*N*_train_×1) along PC*1*,**X** is the binary design matrix of the experiment (*N*_train_×3) with three columns where two columns indicate the experimental conditions (Sc or Se), β is the vector of the weights and intercept constant (3×1), and ε is Gaussian noise with zero mean and unit variance. Regressions were performed for each recording site using 10-fold leave-27-out by random sampling (*N*_*train*_ = 17 trials from the 44 trials leaving out 27) to generate a linear encoding model. At each iteration of the 10-fold linear regression, the prediction value of the encoding model generated for each recording site was assessed using the 27 (left-out) trials by calculating the Pearson’s correlation between the predicted and observed projections. We define the prediction accuracy of a recording site to be the average Pearson correlation across the ten regressions. A recording site ascribed to be of high-encoding performance along PC1 is a recording site with a Pearson’s correlation significantly higher than random distribution (*P* < 0.05, permutation test), generated through conditions swapping repeated over 10,000 iterations.

#### Decoding Performance

Decoding performance was analyzed using groups of recording sites of varying sizes. The groups of recording sites were formed as follows: we first rank-ordered all recording sites based on their encoding performance (i.e., Pearson’s correlation values from highest to lowest). Groups with sizes ranging from 4 to 970 in increments of 2 were formed. For each group being analyzed, we created a subgroup by randomly splitting the studied group in half. The decoding model was then applied to this new sub-group yielding a value of percent-accuracy corresponding to the percentage of successfully decoded trials among the trials in the test data set. This was accomplished in the following manner:

For each trial acquired during sequential stimuli presentation (Seq), the vector ***y***_*Seq*_ was formed where each element is the calculated trial data PC1-projection across the studied subgroup


$${\text{y}}_{\text{Seq}}=\left({\text{y}}_{\text{Seq}}^{1},{\text{y}}_{\text{Seq}}^{2},\ldots ,{\text{y}}_{\text{Seq}}^{N}\right)$$

where *N* is the total number of recording sites in a decoding subgroup. Equivalent vectors **y**_*Scr*_ was formed using trials from scrambled stimuli conditions. Two additional vectors ${\text{y}}_{\text{Seq}}^{\prime }\,\text{and}\,{\text{y}}_{\text{Scr}}^{\prime }$ were generated using linear regression on all the dataset except the tested trial. The purpose here is to calculate for each recording site in the studied subgroup a predicted weight (predicted PC1-projection) for both Seq and Scr experimental conditions. Thus, for a given trial in the test set, the Pearson’s correlation value was computed between either vectors of calculated weights (***y***_*Seq*_ or ***y***_*Scr*_) and the vectors of predicted weights (${\text{y}}_{\text{Seq}}^{\prime }\,\text{and}\,{\text{y}}_{\text{Scr}}^{\prime }$) of the same subgroup sites.

A successful decoding is achieved if the Pearson’s correlation between the calculated weights and predicted weights are higher for trials of the matching experimental condition as opposed to mismatched trial type. This process was repeated for all trials in the test set (12 trials). The decoding performance is measured by the percentage accuracy of decoding corresponding to the percentage of successfully decoded trials among the tested trials. Random decoding performance is 50% due to an equal likelihood of selecting between Seq and Scr condition through random chance. This subgroup analysis was performed 10 times and the average percent-accuracy value was calculated for all thirteen subjects.

#### Cross-Regional Connectivity Analysis

Connectivity between brain regions in response to Seq vs. Scr experimental conditions was examined using granger causality (GC) analysis (Granger, 1969) calculated using an autoregressive (AR) model (Geweke, 1982) provided as part of the Brainstorm software toolbox (Tadel et al., 2011). For each test condition (Se or Sc), GC was calculated for all possible pairs of recording sites. To accomplish this, the recorded time series for each pair was first preprocessed by subtracting the DC offset (the offset is calculated as the mean amplitude from -100 to 100 ms around stimulus onset). The GC metric was calculated for the 100–500 ms response period after the onset of image presentation. For each subject, the GC metric was calculated between each pair of recording sites along with the parametric *p*-value of the mean estimate. For a pair (*x*, *y*), where *x* and *y* represent two studied recording sites, *x* “granger causes” *y* if the GC metric of (*x*, *y*) is higher than (*y*, *x*) with *P* < 0.05 using two Wald statistics according to (Geweke, 1982; Hafner and Hafner, 2008).

The bst_granger function in Brainstorm software toolbox was used to calculate the pairwise GC and associated *p*-value between every electrode site within the same subject. We generated a contingency table by counting the number of electrode pairs that had GC with *p*-value < 0.05 as having significantly high connectivity between brain regions across all subjects, obtaining the tables in Figures 7A,B. Comparison of the distribution of high connectivity regions between Sc and Se groups was performed using the single Chi-Square test in SPSS v. 28 (IBM Inc.). Individual source-sink pair comparisons were independently performed using individual Chi-Square tests that were subsequently corrected for multiple comparisons by computing the adjusted residuals from the Chi-Square test, comparing all possible combinations of pairs. This was performed by computing the adjusted Chi-Square statistics for each combination followed by the adjusted *P*-value. The resulting adjusted *p*-value are plotted in Figure 7C.

**FIGURE 2:**
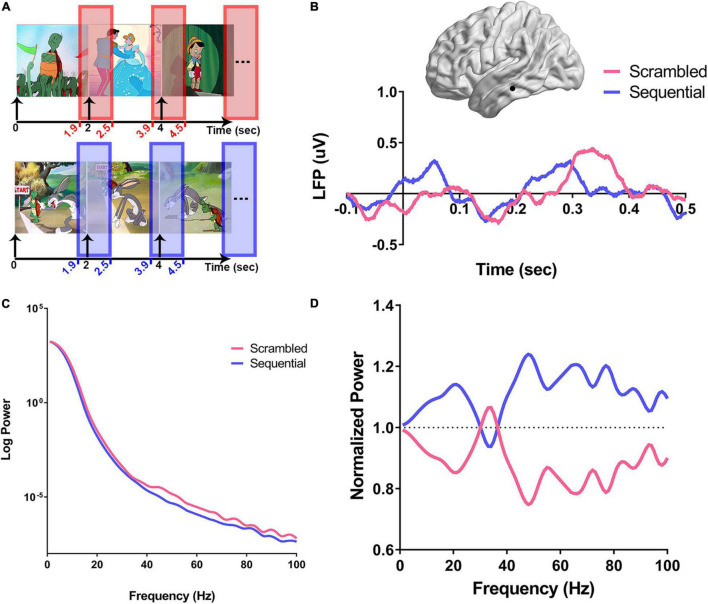
Computation of power spectra in response to scrambled vs. sequential images. For each recording site, the normalized power spectra were calculated as shown in **(A–D)**. **(A)** For each experimental condition (Scr in pink and Seq in blue), the time window around the onset of the image (100 ms before onset to 500 ms after onset of stimulus) was used to calculate the average normalized power spectra. **(B)** An example using a recording site from the left inferior temporal gyrus (ITG) from subject three (S3) is illustrated on a brain surface reconstruction in MNI space. The average variation of the amplitude of local field potentials over time in response to scrambled (pink), and sequential (blue) peri-stimulus onset is shown. Onset of the stimulus is defined as time zero, and all single trials were preprocessed by subtracting a baseline offset calculated by averaging the amplitude of signal for each trial between −0.1 and 0.05 s. **(C)** Peri-stimulus PSDs from the single left ITG recording site are shown in **(B)**. For each trial of the experimental conditions (Scr and Seq), power spectra were derived after *z*-scoring the corresponding time domain signals. The average power spectral density (PSD) for each condition is log-transformed and shows a clear broadband increase over a wide range of frequencies in the power response to Scr vs. Seq. **(D)** Single-trial PSDs are then normalized by dividing by the average of all trials PSDs for the studied recording site, and the average of each condition-specific normalized PSDs is derived.

**FIGURE 3:**
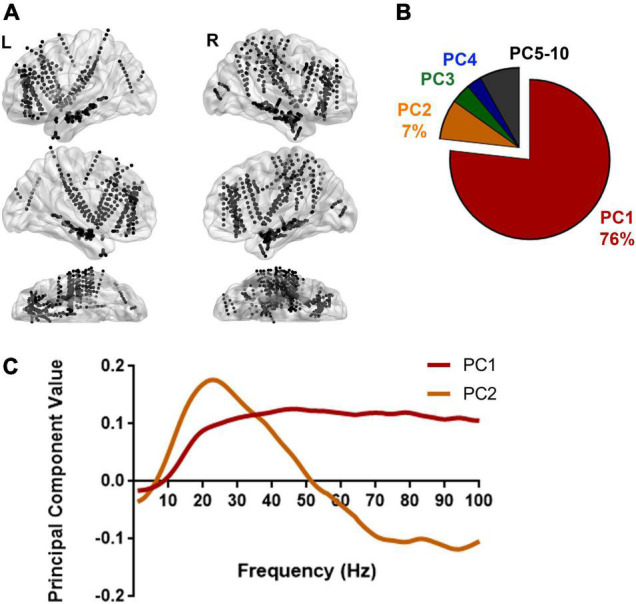
Spectral pattern explaining the variation in power between Scr and Seq images. **(A)** Recording sites from 13 subjects are illustrated on a standard brain surface based on their MNI space coordinates. Each black dot indicates the location of one recording site. L, left hemisphere; R, right hemisphere. Supplementary Table 1 shows the full list of recording sites per subject. By calculating the averaged PSDs for every single recording site illustrated here (*N* = 970 recording sites) using the approach described in Figure 2, a total of 1940 PSDs were used to reveal the spectral variation in response to Scr vs. Seq image sequences. **(B)** Dimensionality reduction using principal component analysis (PCA) on all PSDs yielded the major principal components (PCs) that explain the variance among PSDs. A pie chart shows the distribution of percentage of variance explained by each PC. The primary PC (PC1) explained the majority of variance (76%). **(C)** The top two principal components (PCs) are plotted: PC1 exhibits a broadband variation in spectral power whereas PC2 showed a relative higher power in low frequency range and lower power at higher frequency bands. PCA was also applied to individual subjects and shows consistent patterns for the top PCs (Supplementary Figure 3).

**FIGURE 4:**
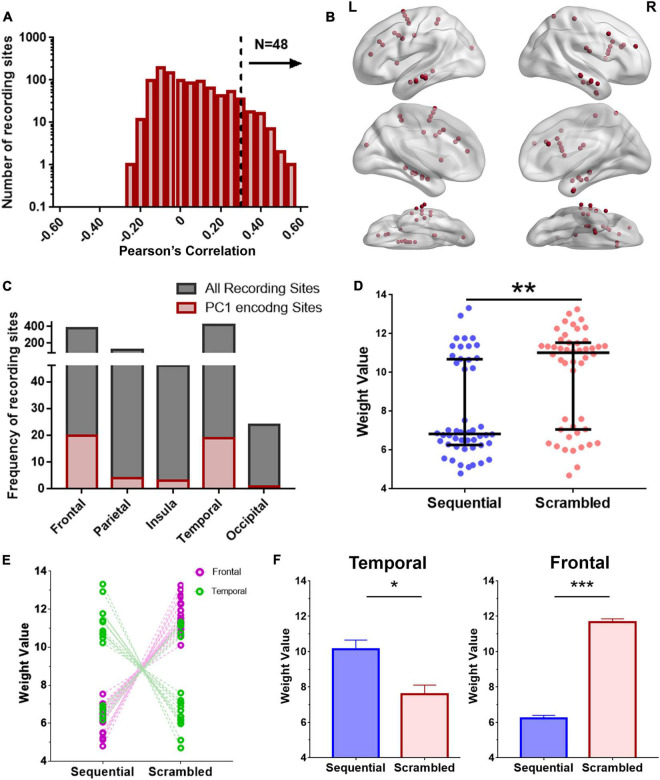
Encoding model for examining regional broadband spectral differences in response to Seq and Scr experimental conditions. **(A)** Histogram of encoding prediction accuracy across all recording sites for PC1 which captures broadband spectral features. High encoding performance recording sites were defined as those with *P* < 0.05 on permutation test (dashed line, Pearson correlation > 0.34). **(B)** Distribution of high encoding performance recording sites (red dots) across all brain regions based on **(A**; *N* = 48 recording sites). *L*, Left hemisphere; *R*, Right hemisphere. **(C)** Distribution of high encoding performance recording sites relative to the total recording sites from each brain region. **(D)** Predicted weights (PC1 projections) for Seq and Scr experimental conditions across the 48 encoding recording sites. Weights from Scr experimental conditions are significantly higher than those of Seq across all sites (^**^P < 0.01, Mann–Whitney test). **(E, F)** Predicted weights in **(D)** were grouped according to temporal vs. frontal locations and also by Seq vs. Scr experimental conditions. There is a significant decrease in temporal broadband power in response to Scr relative to Seq whereas there is a significant increase in frontal broadband power in response to Scr relative to Seq (*P < 0.05 ^***^P < 0.001, Mann–Whitney test). Refer to Supplementary Figure 4 for additional details of the encoding recording sites across subjects, and to Supplementary Figure 5A for further comparison of **(D)** with ISI.

**FIGURE 5:**
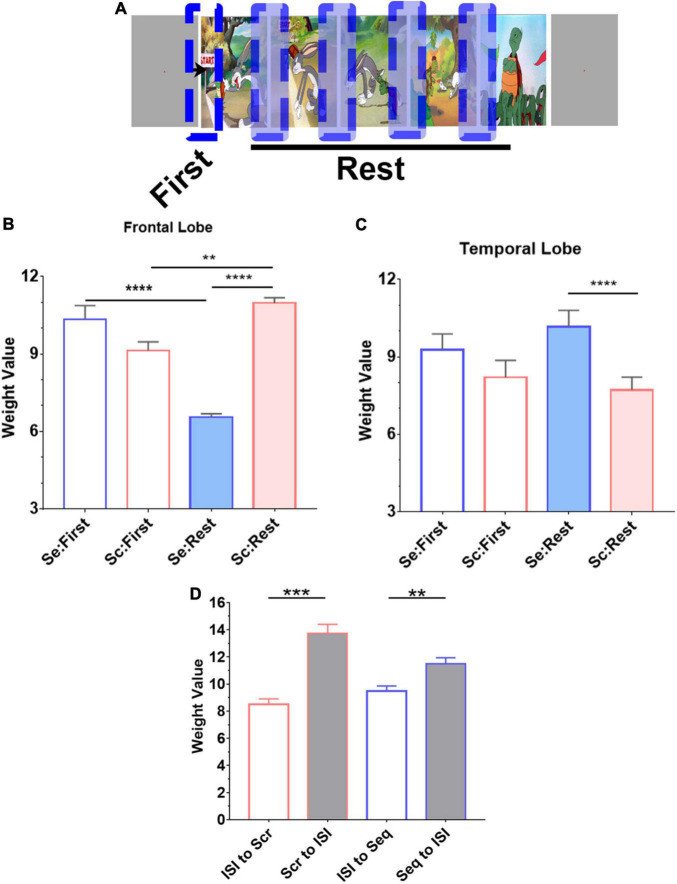
Novelty effect in frontal lobe. **(A)** Trials from both experimental conditions (Seq and Scr) were grouped into two categories based on the location of the image in the story. “First” denotes the first transition in each image series (or story) whereas “Rest” denotes subsequent transitions. **(A, B)** In both temporal and frontal lobes, we observe that the first transition response is not different between the two experimental conditions; there was no significant difference in predicted weights between Scr and Seq in the first transition subset (*P* > 0.8, Kruskal–Wallis test). However, in the frontal lobe **(B)**, the subsequent Seq “Rest” group has a significant decrease in the overall weight compared to the first Seq response, Scr Rest group has a significant increase in the overall weight compared to the first Scr response, and Scr Rest group has a significant increase in the overall weight compared to Seq Rest group (**P < 0.01, ****P < 0.0001, Kruskal–Wallis test). **(C)** In the temporal lobe, the subsequent Seq “Rest” group has an increased overall weights in Seq vs. Scr (****P < 0.0001, Kruskal–Wallis test). **(D)** Mean and SEM of the weight values calculated from the transition between ISI to image presentation during Scr and Seq experimental conditions are significantly different from the transition between image to ISI. There is no difference in the weight values calculated from ISI to Scr or Seq transitions. The responses when transitioning to and from ISI in both Scr and Seq experimental conditions are consistent. (one-way ANOVA with Bonferroni’s multiple comparison test. **P < 0.01 and ***P < 0.001).

**FIGURE 6:**
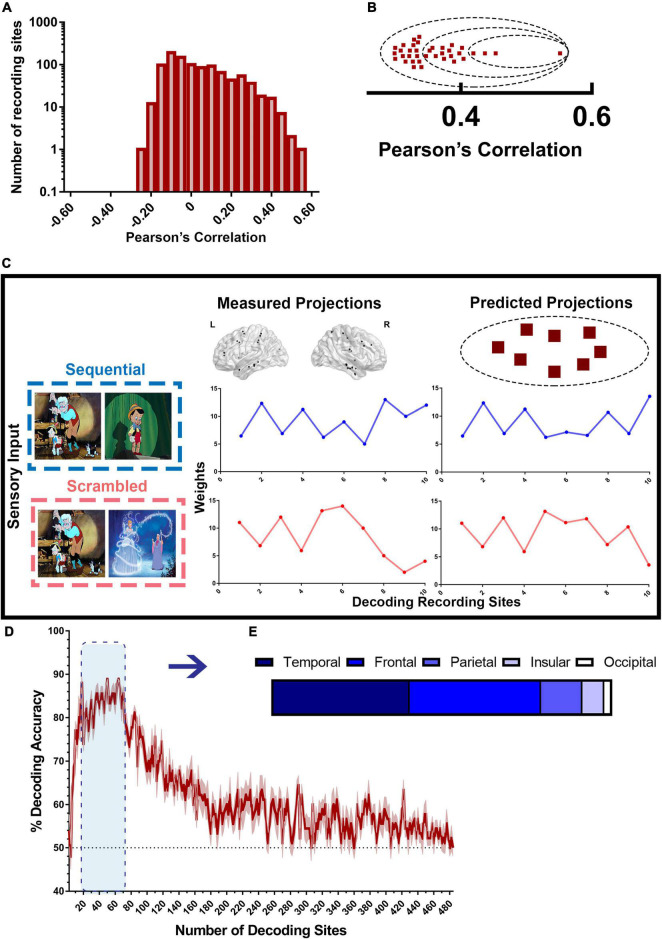
Decoding model for scrambled and sequential patterns. **(A)** Distribution of recording sites ordered by encoding accuracy as shown in Figure 4 used by the decoding model. **(B)** High encoding performance recording sites (red squares) are sorted based on their accuracy from the highest encoding performance to the lowest, then assigned to groups of increasing sizes *n* (dashed circles), containing the *n* highest encoding performance sites. Then, a random subset of half the size of the selected group of sites is used in every iteration of the decoding model. **(C)** illustrates how the decoding model works. To decode the type of narrative from the measured projections, the encoding model for each recording site in the studied group was used to generate “predicted patterns” (right) corresponding to each of the two types of experimental conditions. The set of measured projections from a subset of 10 recording sites from both Scr (red) and Seq (blue) is compared to the predicted projections for each stimulus from the same sites. Each of the two predicted patterns was then correlated with the measured pattern. If the predicted pattern corresponding to the correct stimulus type had the highest correlation with the measured pattern, decoding is considered successful for that single trial. **(D)** Decoding accuracy of Scr vs. Seq conditions using broadband spectral pattern of various sizes and subsets of sites. The accuracy (mean ± SEM) for varying size of groups of sites is shown. The dashed black line represents random chance (50%). **(E)** Distribution of sites with the highest decoding accuracy across brain regions.

**FIGURE 7:**
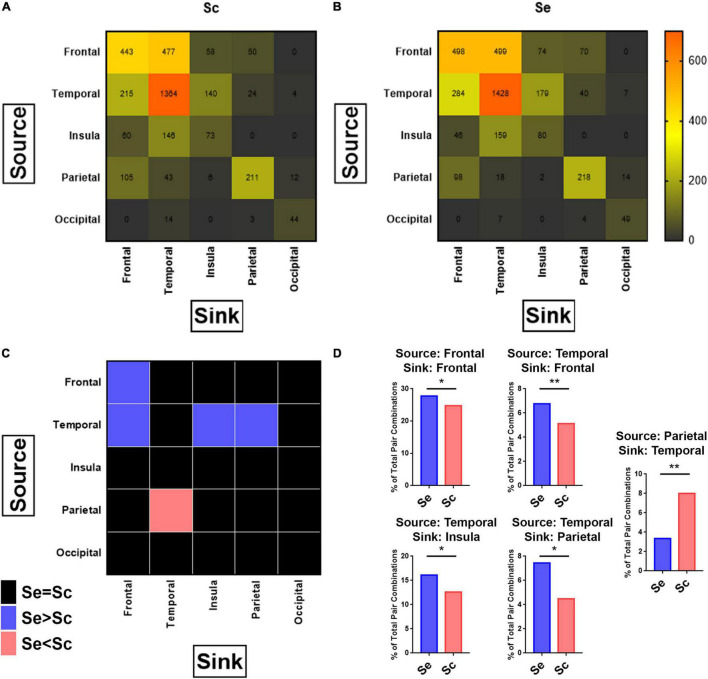
Connectivity between brain regions in response to sequential and scrambled patterns. For each combination of possible pairs of recording sites per subject, granger causality was applied to calculate the connectivity between the studied pairs along the first 100–500 ms after the onset of the images corresponding to sequential and scrambled patterns independently (see section “Materials and Methods”). **(A, B)** Heatmap of the number of pairs with high connectivity between sources (each row represents one source brain region), and sinks (each column represents one sink brain region) for scrambled **(A)**, and sequential **(B)** patterns. Chi-Square test for comparing the distribution of high connectivity regions between Scr vs. Seq shows a significant difference in the distribution. Pearson’s Chi-Square: 193.1, *P* < 0.001. **(C)** Heatmap of *p*-values after applying the Chi-Square test to determine which source-sink brain regions exhibit significant differences in the number of connections between sequential and scrambled patterns. In pink are the source-sink cells that has Seq < Scr whereas in blue are source-sink cells with Seq > Scr for *P* < 0.05. The nonsignificant differences are colored black. **(D)** Re-demonstration of findings from **(C)** showing that sequential patterns has a significantly higher percentage of connectivity pairs compared to scrambled patterns when the (source, sink) combinations are: (Frontal, Frontal), (Temporal, Frontal), (Temporal, Insula), (Temporal, Parietal), and scrambled patterns has a higher percentage of connectivity pairs compared to sequential patterns when the (source, sink) combination is (Parietal, Temporal). Chi-Square test. *P*-value adjusted for multiple comparisons based on adjusted residuals: *P < 0.05, **P < 0.01. See also Supplementary Figure 6.

### Statistical Analysis

Statistical analysis was performed using MATLAB 2019a (Mathworks, MA, United States) and Graphpad Prism 8 (GraphPad Software, CA, United States). The Fisher’s exact test (Fisher, 1935; Pitman, 1937) was used to determine recording sites with high encoding performance by comparing the value of Pearson’s correlation to that of a random distribution. Unless otherwise stated, statistical significance was set at α < 0.05.

### Materials Availability

Datasets and analysis code are available from the corresponding author upon reasonable request. Further information and requests for resources will be fulfilled by the corresponding author, Nicholas AuYong (nicholas.au.yong@emory.edu).

## Results

### Subjects Cohort and Visual Task

Thirteen adult subjects (6/13 females, mean age 35 years) undergoing intracranial electrode monitoring for clinically indicated evaluation of epilepsy participated in these experiments. In total, 114 electrodes were implanted for all 13 subjects, providing 970 recording sites. 300 recording sites were excluded as described in the section “Materials and Methods.” All subjects participated in the same visual task that involves viewing colored cartoon images from seven common fables (with five images per story). Images were presented in a scrambled pattern (Scr) during the first part of the experiment and then in sequential order (Seq) during the second part. Example image sequences as viewed by subjects is shown in Figure 1 (see Supplementary Figures 1, 2 for full sets of Scr and Seq images). Each image was presented without repetition for 2 s. For the Seq experimental condition, the last image from one story and the first image of the next was separated by a gray screen, displayed for 6 s.

### Scrambled and Sequential Patterns Are Associated With Broadband Spectral Power Variations

Prior human surface EEG studies described the “N400 Effect,” consisting of a negative-going deflection that peaks in the frontal LFP around 400 ms, evoking a larger amplitude in response to scrambled sequence of images than to coherent narrative sequences (Cohn et al., 2012). Based on these findings, we carried out our post-stimulus response analysis up to 500 ms following image presentation (Figure 2A). Time domain LFP analysis was performed after baseline signal correction by subtracting the average amplitude between -100 and 50 ms around stimulus onset. An illustrative example from the left inferior temporal gyrus (ITG) of subject three (S3) is shown in Figure 2B demonstrating a clear distinction of Scr and Seq responses between 300 and 500 ms from stimulus onset (at 0 s). This result is analogous to the source localization of N400 reported by Kutas and Federmeier (2000) to be originating in the left ITG. The *z*-scored normalized time domain signals of the individual trials were then used to construct the peri-stimulus PSDs for studying the distinction between the two experimental conditions in the frequency domain. Figure 2C shows a single subject example of the average PSDs from all trials within the ITG of S3 for each condition (Scr vs. Seq), demonstrating a clear broadband power shift between Scr and Seq subsets. This broadband modulation is distinct after normalization of all PSDs (Figure 2D, see section “Materials and Methods”).

The spectral features associated with cerebral processing can exhibit *“narrowband patterns,”* reflecting an underlying synchronized neuronal population activity resulting in power modulation over a narrow frequency range (Eckhorn et al., 1988; Fries et al., 2008; Akam and Kullmann, 2014; Hermes et al., 2015) and/or a “*broadband pattern*” as a result of asynchronous neuronal firing yielding an increase in spectral power across a broad range of frequencies (Winawer et al., 2013; Hermes et al., 2015; Fasoli et al., 2018; Sabra et al., 2020). To further investigate if either narrowband or broadband spectral features predominate during Scr/Seq stimuli presentation, PCA was applied to determine the frequency bands at which the most variance in spectral power between Scr and Seq experimental conditions occur. Normalized PSDs were grouped according to the stimulus condition associated with each trial (Seq vs. Scr), and then averaged within-group. This resulted in two normalized mean PSDs (one for Seq and one for Scr) for each recording site as illustrated in Figure 2D. PC analysis was then performed on pooled data from all subjects and recording sites (Figure 3). We found that the first PC (PC1) accounted for 76% of variance in spectral power (Figure 3B) while the second PC (PC2) accounted for 7% of the variance. When examined in the frequency domain, the variation of PC1 exhibited a distinct broadband profile with elevation in power across a wide range of frequencies (>10 Hz; Figure 3C). The observed PC1 pattern (Figure 3C) closely mimics the pattern of power variation observed in Figure 2D. PCA results demonstrate that the difference in neuronal response to Seq vs. Scr sequences across all recording sites/subjects is better explained by a broadband shift in power rather than a narrowband oscillation pattern. To further determine the robustness of this finding, we performed a similar PCA over individual subjects showing conserved broadband motif of the highest performing PC (i.e., PC1; Figure 3-A). However, PCA applied to single-subject data revealed some inter-subject variation in the pattern of PC2 across subjects (Figure 3-B). Since the majority of variance was explained by PC1, we focused our subsequent analyses on the first PC. The conservation of broadband motif when the analysis is applied to each subject separately rules out the possibility that the broadband is driven by one or few brain regions or subjects.

### Broadband Spectral Features Encode Information About the Visual Sequences

The degree in which broadband spectral features encode information about the visual stimuli (Seq vs. Scr) was independently estimated using a *PC-projection* encoding model, applied to each recording site. For each recording site, an encoding model was derived and used to predict the experimental condition (Seq vs. Scr) based on the calculated PC1 weight. We calculated the predictive weight for each of the two experimental conditions as described in the section “Materials and Methods”. Then we calculated the Pearson correlation between the model’s predictions and the observed spectral power along PC1 across a held-out validation dataset (Figure 4A).

A recording site is considered to be of high encoding performance if the correlation exceeds a predefined threshold (Pearson correlation > 0.34 based on *P* < 0.05 on permutation test). Recording sites with high encoding performance over the broadband PC1 were distributed widely across the brain and were identified in the temporal, prefrontal, occipital, insular and parietal cortices (Figures 4B,C). Recording sites with high broadband encoding performance models were present in twelve of the thirteen subjects (Supplementary Figures 4A,B). The predicted broadband weight values of Scr and Seq experimental conditions are plotted for the recording sites with high encoding performance (Figure 4D). The results show that the Scr experimental condition has significantly higher weights than the Seq experimental condition [*P* = 0.01; Kolmogorov–Smirnov test (Marsaglia et al., 2003)]. When assessing recording sites for different brain regions, we observed a distinct pattern of weight values for Scr and Seq experiments conditions. We found significantly higher PC1 weights in Seq vs. Scr among temporal sites compared to significantly lower weights in Seq vs. Scr among frontal sites (Figures 4E,F). Most of the encoding recording sites in the temporal lobe are localized in the ventral temporal pathway (2 in the fusiform, 15 in the inferior temporal gyrus and 2 in the middle temporal gyrus). The results shown in Figure 4D were significantly different from the broadband projections of the response to gray screens or inter-stimulus interval (ISIs) between the stories (Supplementary Figure 5).

### Regional Specialization of Broadband Spectral Encoding for Novelty Effect Within the Frontal Lobe and for Narrative Coherence Within the Temporal Lobe

We evaluated single trial projections comparing the first transition in an image series to the subsequent transitions to determine how the brain regional spectral features are altered during sequential vs. scrambled test conditions within the frontal and temporal lobes (Figure 5). In the frontal lobe (Figure 5B), we observed a dramatic reduction in broadband power with subsequent trials when they are part of a sequential visual narrative. In contrast, images displayed in a scrambled pattern resulted in an increase in broadband power. The PC1 weights were similar between Seq and Scr on the first transition, but these weights were significantly lower in Seq vs. Scr with subsequent transitions within the same image series. Taken together, these findings indicate that frontal lobe broadband spectral response habituates to sequentially presented images but not to scrambled patterns reflecting the persistent novelty effect in the scrambled pattern. However, in the temporal lobe (Figure 5C), we observed a dramatic reduction in broadband spectral power with subsequent trials if they are part of a scrambled pattern but no change in power when images are displayed in a sequential visual narrative sequence. In the frontal lobe, we observed that PC1 weights were similar between Seq and Scr on the first transition. However, these weights were significantly higher in Seq vs. Scr in subsequent transitions within the same image series. This indicates that the broadband response adapts to the Scr sequence of images but not to Seq. The first Seq and Scr transitions represent the transition from ISI (i.e., gray screen) to image. For each of the two conditions, the transition from ISI to image was significantly different from the transition from image to ISI (Figure 5D).

### Visual Narrative Stimuli Are Accurately Decoded Using Broadband Spectral Features

To test the accuracy of broadband spectral features in classifying the type of visual narrative stimuli, we applied a model-based decoding analysis (described in the section “Materials and Methods” and in Figure 6) using PC1 weights (or projections) that captures broadband spectral features. The accuracy of this model in distinguishing between scrambled patterns and sequential visual narrative stimuli compared to random chance (50%; since we are classifying between two experimental conditions) was tested. Multiple iterations of the decoding scheme were constructed from data recorded using a growing subset of recording sites in the order of decreasing encoding accuracy. An overall decoding accuracy of 86–90% was attained in distinguishing Seq vs. Scr patterns using 20–70 recording sites in the order of decreasing encoding accuracy. These recording sites include random combinations from the top 140 encoding sites (see section “Materials and Methods”) and were predominantly located in the temporal (40%) and frontal (39%) regions like the pattern observed in the encoding model (Figure 6E). As a result, this finding suggests that information about the visual narrative can be accurately decoded by the broadband spectral features, largely from these two brain regions (Figure 6D).

### Visual Narrative Stimuli Modulate Temporal-Parietal-Insular-Frontal Connectivity

Granger causality as described in (Granger, 1969; Geweke, 1982 #20) was employed to examine if interregional connectivity between all studied brain regions changes in response to Seq and Scr experimental conditions. A source-sink couplet between each site was identified as described in the section “Materials and Methods”, and the number of source-sink couplets between different regions is shown in Figures 7A,B. When comparing Seq vs. Scr, we observed a significantly higher proportion of source-sink couplets within the frontal lobe, and also in the couplets connecting temporal to frontal, temporal to insular, and temporal to parietal cortices in the listed direction. A significantly lower proportion of source-sink couplets connecting parietal to temporal cortices was found (Figure 7C). These findings along with those of Figures 4, 5 indicate that a sequential visual narrative involves increased temporal input to the frontal, insular and parietal cortices leading to suppression of a broadband spectral response in the frontal lobe.

To further determine if differences in the number of source-sink couplets between Seq vs. Scr (Figure 7D) is also associated with modulation in connectivity coefficient, we examined the distribution of connectivity coefficient for each source-sink regional couplets (Supplementary Figure 6). For all couplets that showed significant increase or decrease in the number of connectivity pairs between Seq and Scr, we observed an overall respective increase or decrease in the number of pairs without a change in average connectivity coefficients. For each pair showing significant difference between Seq and Scr, we compared the amount of connectivity in Seq and Scr to the connectivity during ISI of the corresponding conditions and for all pairs the connectivity during Seq and Scr were significantly higher than ISI.

### Lateralization in the Connectivity Modulation

Interregional connectivity between cortical areas was found to be differentially modulated during Seq and Scr conditions. There is significantly higher connectivity between the left temporal and the left frontal lobe during Seq image viewing compared to Scr conditions (Fisher’s exact test *P* < 0.001). In contrast, viewing Scr images resulted in increased connectivity between the right parietal and right temporal lobes compared to Seq testing conditions (Fisher’s exact test *P* = 0.0083). Lateralization was absent for the other brain regions.

## Discussion

### Broadband Spectral Power Has High Prediction Value for Deciphering Seq and Scr Conditions

Decoding of Seq and Scr patterns using broadband spectral pattern was achieved with increased accuracy in sites with highest encoding value from the temporal, frontal, parietal, and insular cortices. Temporal, frontal, and parietal lobes were previously shown to exhibit different responses to Seq vs. Scr equivalent conditions in the time domain (Cohn, 2014; Cohn and Kutas, 2017; Isik et al., 2018). Our results show that the broadband component in the spectral domain contains the requisite information to accurately decode what type of image patterns are being observed. While an encoding-decoding model approach does not precisely reveal how the brain processes visual narrative information, the high-performance based on broadband spectral features alone suggests that broadband activity is highly informative about image sequence type and perhaps the underlying cognitive process as well.

### Encoding of Visual Sequence Is Mainly Explained by a Broadband Modulation of Spectral Power

Cognitive processing of visual narrative provides insight into how the brain interprets the continuous barrage of visual information, and other types of sensory information, encountered moment to moment. Comprehension of visual narrative is highly dependent on the structural and semantic coherence between images (Cohn et al., 2012). The greater the semantic discontinuity between images, the harder it is to make connections between them (i.e., bridging inference; Saraceni, 2000, 2001; Magliano et al., 2016). In the cognition of visual narrative stimuli, the perception of the continuity across scenes may rely on a “the continuity constraint” as proposed by Cohn (Cohn, 2020c); where the link is made for the representation of objects in each frame to refer to the same thing (e.g., the picture of a rabbit in each frame is commonly recognized as Bugs Bunny across all the frames). This is similar to the concept of “mapping” proposed by Loschky et al. (2020) that links upcoming frames to the preceding ones. Accordingly, a viewer maps incoming information in working memory if the flow of information is coherent, and builds upon this foundation. Mapping requires the monitoring of factors affecting the continuity of events such as time, space, and causality. On the other hand, in scrambled conditions, this continuity is interrupted between the adjacent frames and the characters and scenes are continuously changing between them. This creates a perception that they are contextually novel despite familiarity with the content of the images themselves.

Our present study further clarifies how distinct brain areas participate in this cognitive process using sequential and scrambled visual sequences. We first demonstrate that variation in broadband spectral power can distinguish between Scr and Seq conditions. Broadband encoding in the human brain has been previously implicated in encoding different sensory modalities and is thought to reflect the local de-synchronous neural response of underlying neuronal circuitry to varying stimulus conditions (Manning et al., 2009; Hermes et al., 2015). Sites encoding sequential and scrambled conditions represented 3% of the studied sites, but were preferentially spanning the frontal and temporal lobes. The distribution and percentage of the encoding sites are consistent with what was previously reported when using a comparable design (Isik et al., 2018).

Saccadic effect was previously reported to be executed at the level of the cortex by the frontal eye field and supplementary eye filed regions of the prefrontal cortex (Petit et al., 1995; Lobel et al., 2001; Lachaux et al., 2006), and are believed to drive broadband spectral effect in the anterior temporal lobe (Katz et al., 2020), and in early visual areas including V1 and V2 (Kern et al., 2021). In our study, these brain regions were largely not recorded from. Thus they were not primary to our analysis and did not contribute much to the PCA.

### Regional Specialization of Responses to Seq and Scr Images

A closer look at the Seq and Scr responses in the frontal and temporal lobes revealed regional specialization of responses. The sites in the frontal lobe responded with increase in broadband power in response to novel or unexpected (Scr) scenes and decrease in power in response to expected (Seq) images within a coherent narrative. The temporal lobe sites, however, responded with broadband power increase to expected chronological flow of Seq images and decrease in power to the unexpected sequence of Scr scenes. These contrasting frontal and temporal responses reinforce our current understanding of their distinct roles in detecting novel events vs encoding episodic memory, respectively (Ranganath and Rainer, 2003; Petrides, 2007; Schomaker et al., 2020).

Our findings show that sites within the temporal lobe exhibit broadband spectral power increase while encoding Seq experimental conditions where panel-panel transitions have high semantic and structural coherence. The engagement of the temporal lobe, and more specifically the ventral temporal pathway, in the encoding of Seq images is consistent with its specialized role in object recognition (Penfield and Perot, 1963; Goodale and Milner, 1992; Allison et al., 1999; Kreiman et al., 2000; Joseph, 2001) and encoding episodic memories that involves recognition of seen objects and integration of sequential events (Ranganath and D’Esposito, 2005; Sederberg et al., 2007). Novelty detection is a cognitive process that involves temporal sensory integration that assesses the expectedness of a stimuli in the perceived setting. In other words, it is the brain’s derived likelihood of perceiving a stimulus given the preceding flow of events (Berns et al., 1997) to determine if a stimulus is either salient, new, and/or unexpected.

Novel visual stimuli processing has been reported in the orbitofrontal cortex of macaque where neurons responded to novel never seen before visual images but not to familiar images (Rolls et al., 2005). In humans with frontal lobe injuries (Daffner et al., 2000), EEG response to novel stimuli in the frontal lobe at the level of P3 was reduced and attributed to the disruption of directed attention to novel objects after frontal lobe damage. Our results confirmed the same effect in frontal intracranial electrodes where broadband response to scrambled images was higher than that observed for sequential images. Furthermore, we investigated if an initially encountered image produces a response similar to that of a random image in the frontal lobe, as they are both equally surprising, and both represent the phase of “laying the foundation,” where the first node is built as a basis for the expectations regarding the upcoming events (the spatiotemporal framework and context of events; Cohn, 2020c; Loschky et al., 2020). Our results confirm the response to scrambled presentation of images and that to the first image of a sequential pattern is no different. Our results are analogous to those reported by Cohn et al. (2012) regarding the similarity of the response to the first images in sequential and scrambled conditions using ERP for N400 collapsed across the frontal, central frontal, and central temporal electrodes.

Together, our findings suggest that the cognition of visual narrative may involve both novelty and temporal processing *via* an intricate network of frontal-temporal signaling (Tiitinen et al., 1994; Petrides, 2007; Cohn, 2014; Miller et al., 2015; Schomaker et al., 2020).

### Alteration of Interregional Connectivity in Response to Image Sequences

**FIGURE 8:**
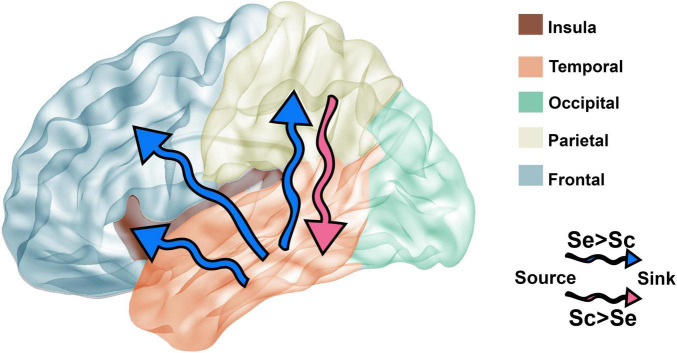
Comparison of brain connectivity dynamics between Seq and Scr stimulus. Based on the connectivity results presented in Figure 7, the directionality of the connectivity between brain regions is affected by the type of stimulus presented. Blue arrows represent the connectivity with significantly higher number of connections between source and sink during Seq, whereas the pink arrow represents the connectivity with significantly higher number of connections between source and sink during Scr. Connectivity from the temporal lobe to destinations: insular, parietal, and frontal lobes is higher during Seq vs. Scr (blue arrows). The parietal lobe has higher connectivity to the temporal lobe during Scr vs. Seq (pink arrow).

A further look at the dynamics of interregional connectivity (Figure 8) revealed that during sequential presentation of images, the temporal lobe leads the flow of information of the frontal, insular, and parietal lobes. However, during the presentation of scrambled images, the parietal lobe response leads that of the temporal lobe. These results show that in addition to the regional specialization in the neural response to the type of visual patterns, there are also temporal alterations to interregional connectivity according to the narrative coherence between visual images. As such, the network dynamics of visual coherence processing both within and across brain regions changes with respect to the sequence type.

Our method of assessing connectivity was based on studying how much two recording sites are temporally related; the ability to predict a time domain response to a stimulus of a site (sink) based on a precedent response observed in another site (source). This connectivity metric calculated using GC evaluates the directionality of the flow of information between two brain regions at the network level but it doesn’t clarify if the dynamic of neuronal activity is driven by excitatory or inhibitory neurons.

### Pertinence of Narrative Processing in Non-visual Sensory Modalities

The study of visual narratives is an important emerging field in cognitive neuroscience (Cohn, 2020c) as the underlying semantic processing network may support semantic processing across multiple sensory modalities (Kutas and Federmeier, 2000; Ralph et al., 2017; Manfredi et al., 2018; Cohn, 2020a). On surface EEG, an anterior “N400” engagement ERPs response is a characteristic feature during processing scrambled sequence of static (Cohn, 2014; Cohn et al., 2014; Cohn and Kutas, 2017) and dynamic visual scenes (Sitnikova et al., 2008). Interestingly, the N400 effect is comparable with findings during language comprehension paradigms when comparing brain response of unrelated/related consecutive words (Bentin et al., 1985), incongruent/congruent sentences and true/false sentences (Kutas and Hillyard, 1980). Thus, the N400 response seems to be modality-independent and closely associated with semantic processing. In this work, we add a new dimension to our understanding of the cognitive processing of visual narrative by demonstrating the underlying broadband nature of sensory signal processing that involves temporally distinct temporo-frontal connectivity. These findings provide the needed evidence from in-vivo intracranial human data that will guide single-neuron recording studies to further clarify the cellular mechanism subserving visual narrative processing. Future testing with different sensory modalities may further elucidate the potentially shared mechanisms across sensory modalities. Moreover, future analysis could investigate study if narrative processing mechanisms are affected in certain brain conditions including dementia, autism spectrum disorder, ADHD, and schizophrenia (Coderre, 2020; Cohn, 2020b).

### Lateralization of Interregional Connectivity

The left-hemispheric lateralization in the modulation of fronto-temporal connectivity during Seq vs. Scr conditions is analogous to the reported EEG lateralization of the response to scrambled sequences (Cohn et al., 2012), and to the fronto-temporal left lateralization of the connected speech (Neville et al., 1991; Tyler and Marslen-Wilson, 2008; Peelle, 2012). This result further supports the argument that narrative processing in the brain could be modality independent. The right-hemispheric lateralization of the increase in parieto-temporal connectivity during Scr vs. Seq image presentations is in agreement with the involvement of the right parietal in spatio-visual perception of objects (Goodale and Milner, 1992). To our knowledge this is the first intracranial study that demonstrates lateralization in the processing of visual narrative in the human brain.

### Potential Confounding Factors

A potential confounding factor is that images as part of a visual narrative have higher spatial coherence (Malcolm et al., 2016) and may contribute to the differing brain responses between Seq and Scr conditions. Spatial coherence is an important feature in narrative congruency. Yet, when images are presented in the narrative’s chronological order, they exhibit abrupt content changes including location, clothing, and characters while preserving the semantic and structural coherence.

Moreover, it could be argued that the observed difference between Scr and Seq responses could be the result of reduced attention or cognitive disengagement when images are presented in Scr order. To address this issue, we compared the responses during ISIs (i.e., first 4 s of gray screen presentation) during Scr and Seq conditions at different time windows. Responses during ISI during Scr and Seq testing conditions were not significantly different for 99% of the sites. Furthermore, the handful sites that had significantly different Scr vs. Seq ISIs were not among the encoding sites.

Another factor that may explain the difference in response between Scr and Seq stimuli is the common characters across sequential as compared to scrambled image patterns containing different characters. Although the same argument for spatial coherence factor can be applied here, we want to emphasize an additional point; our encoding model uses the average of the broadband response to Seq vs. Scr conditions to calculate the encoding accuracy of a site, which levels out the effect attributed to the difference between image components and focuses the analysis on the differences attributed to the articulation between the images.

Finally, it could be argued that the observed responses are a product of the luminance and chromatic differences (i.e., colors and brightness) between image panels. Our experiment doesn’t eliminate the possible integration of these parameters and others during the processing of visual narrative. However, the analyzed recording sites are mainly distributed across brain regions in high cognitive areas and not in primary visual areas that encode luminance and chrominance.

### Brain Network Spectral Analysis Limitations

In this study, we chose to focus on spectral analysis to identify key differences in brain regional responses when viewing sequential or scrambled images. We selected our time window according to previously reported time-to-peak responses to visual stimuli, which on average is within 200–400 ms range post image onset (Rolls et al., 2005; Kishiyama et al., 2009; Kumaran and Maguire, 2009; Axmacher et al., 2010; Davachi and DuBrow, 2015; Isik et al., 2018; Miller et al., 2015). While time domain analysis, including time-to-peak investigations at the brain network level can be a powerful technique (Kucyi et al., 2020), we opted to forgo such analysis in this study due to variation in the implanted location and number of electrodes in each subject and across subjects.

The use of a common average reference over a small number of electrodes is unlikely to be fully inert. However, we have previously shown that similar analysis using a common average reference was not affected by the small number of electrodes, even with individual PCA analysis carried out for each subject separately (Sabra et al., 2020). Common average referencing is not infrequently employed in sEEG studies (Kubánek et al., 2009; Gaona et al., 2011; Schalk et al., 2017) although we do recognize that other sEEG reference schemes exists, albeit with limited evidence as to how one should select among them (Li et al., 2018).

## Data Availability Statement

The raw data supporting the conclusions of this article will be made available by the authors, without undue reservation.

## Ethics Statement

The studies involving human participants were reviewed and approved by Institutional Review Board at the Medical University of South Carolina. Written informed consent to participate in this study was provided by the participants or participants’ legal guardian/next of kin.

## Author Contributions

ZS and TN: conceptualization. ZS, TN, and NA: methodology. ZS: software and investigation. ZS, AA, TN, and NA: formal analysis. TN, LB, and NA: resources. ZS, AA, TN, LB, and NA: writing—original draft and writing—review and editing. ZS and AA: visualization. TN and NA: supervision, project administration, and funding acquisition. All authors contributed to the article and approved the submitted version.

## Conflict of Interest

The authors declare that the research was conducted in the absence of any commercial or financial relationships that could be construed as a potential conflict of interest.

## Publisher’s Note

All claims expressed in this article are solely those of the authors and do not necessarily represent those of their affiliated organizations, or those of the publisher, the editors and the reviewers. Any product that may be evaluated in this article, or claim that may be made by its manufacturer, is not guaranteed or endorsed by the publisher.
