# Supplementary material for: Modulation of Spectral Representation and Connectivity Patterns in Response to Visual Narrative in the Human Brain
Source: Front Hum Neurosci. 2022 Oct 6;16:886938. doi: 10.3389/fnhum.2022.886938 (PMC9582122; doi:10.3389/fnhum.2022.886938)
Supplement: Supplementary file 1 [file Table_1.docx]

## Supplementary Tables

| ID | Electrodes Location | Gender | Age | Nb. Contacts  (Studied/Total) | Epileptic zone |
| --- | --- | --- | --- | --- | --- |
| 1 | Right: Frontal, insular, parietal, temporal | Female | 25 | 69/80 | Right parahippocampus and hippocampus |
| 2 | Right: Frontal, parietal, temporal | Female | 66 | 70/76 | Right postcentral gyrus |
| 3 | Left: Frontal, insular, parietal, temporal.  Right: Temporal, occipital | Male | 30 | 109/130 | Left and right hippocampi |
| 4 | Left: Temporal, occipital.  Right: Occipital | Male | 32 | 45/70 | Left hippocampus and left occipital |
| 5 | Left: Temporal | Female | 41 | 18/40 | Left hippocampus and anterior temporal lobe |
| 6 | Bilateral: Frontal, insular, parietal, temporal | Male | 14 | 96/140 | Left and right hippocampi, right temporal, and right frontal |
| 7 | Right: Frontal, parietal, temporal | Male | 21 | 62/124 | left pre-central, post-central, and supramarginal |
| 8 | Bilateral: Frontal, insular, parietal, temporal | Female | 46 | 122/150 | Left hippocampus and temporal lobe |
| 9 | Left: Frontal, insular, parietal, temporal | Male | 21 | 81/90 | None of the electrodes |
| 10 | Bilateral: Temporal | Female | 62 | 52/80 | Left and right hippocampi |
| 11 | Bilateral: Frontal, insular, parietal, temporal | Female | 42 | 117/140 | Right hippocampus |
| 12 | Right: Frontal, insular, parietal, temporal | Male | 37 | 66/80 | Right hippocampus |
| 13 | Left: Frontal, insular, parietal, temporal | Male | 21 | 63/70 | Left hippocampus |

**Supplementary Table 1. List of participants and electrodes location, Related to Fig. 3**. Distribution of 970 studied recording contacts across participants and brain regions that met inclusion criteria in proportion to the total recording contacts. All subjects were implanted with depth electrodes, except S2, who had an extra implanted subdural grid covering the mentioned regions. In general, “Frontal” electrodes traverse the frontal cortex into deeper or medial structures within the frontal lobe. “Temporal” electrodes traverse the temporal cortex and land into the amygdala or hippocampus. “Insular” electrodes extend from the insula to the frontal or parietal cortices. “Occipital” electrodes extend from medial occipital regions to the occipital cortex. Epileptic zone is mentioned in the last column and excluded from all analysis.
