# Supplementary figures and images for: Modulation of Spectral Representation and Connectivity Patterns in Response to Visual Narrative in the Human Brain
Source: Front Hum Neurosci. 2022 Oct 6;16:886938. doi: 10.3389/fnhum.2022.886938 (PMC9582122; doi:10.3389/fnhum.2022.886938)

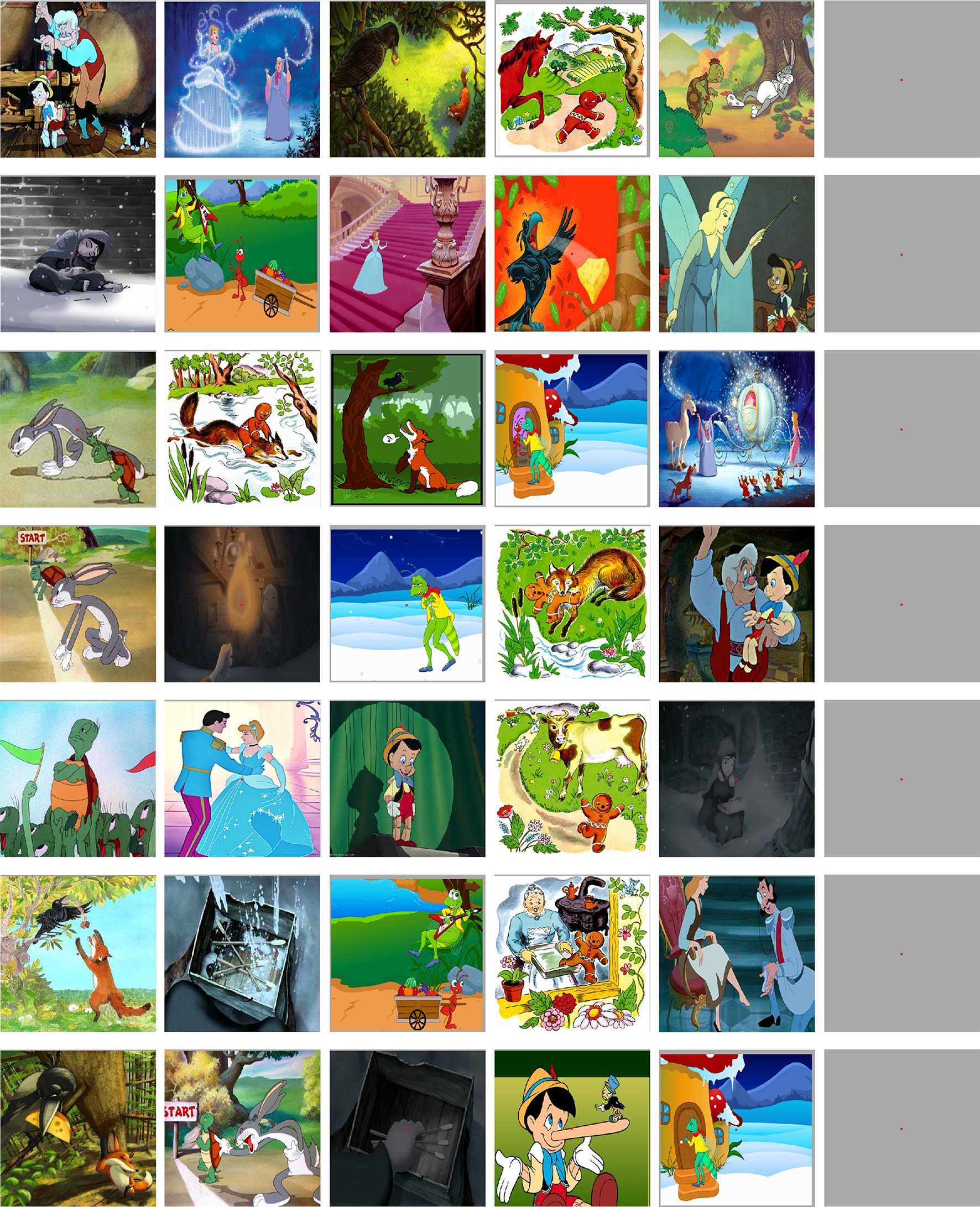

Supplement: Supplementary file 2 [file Image_1.TIF]

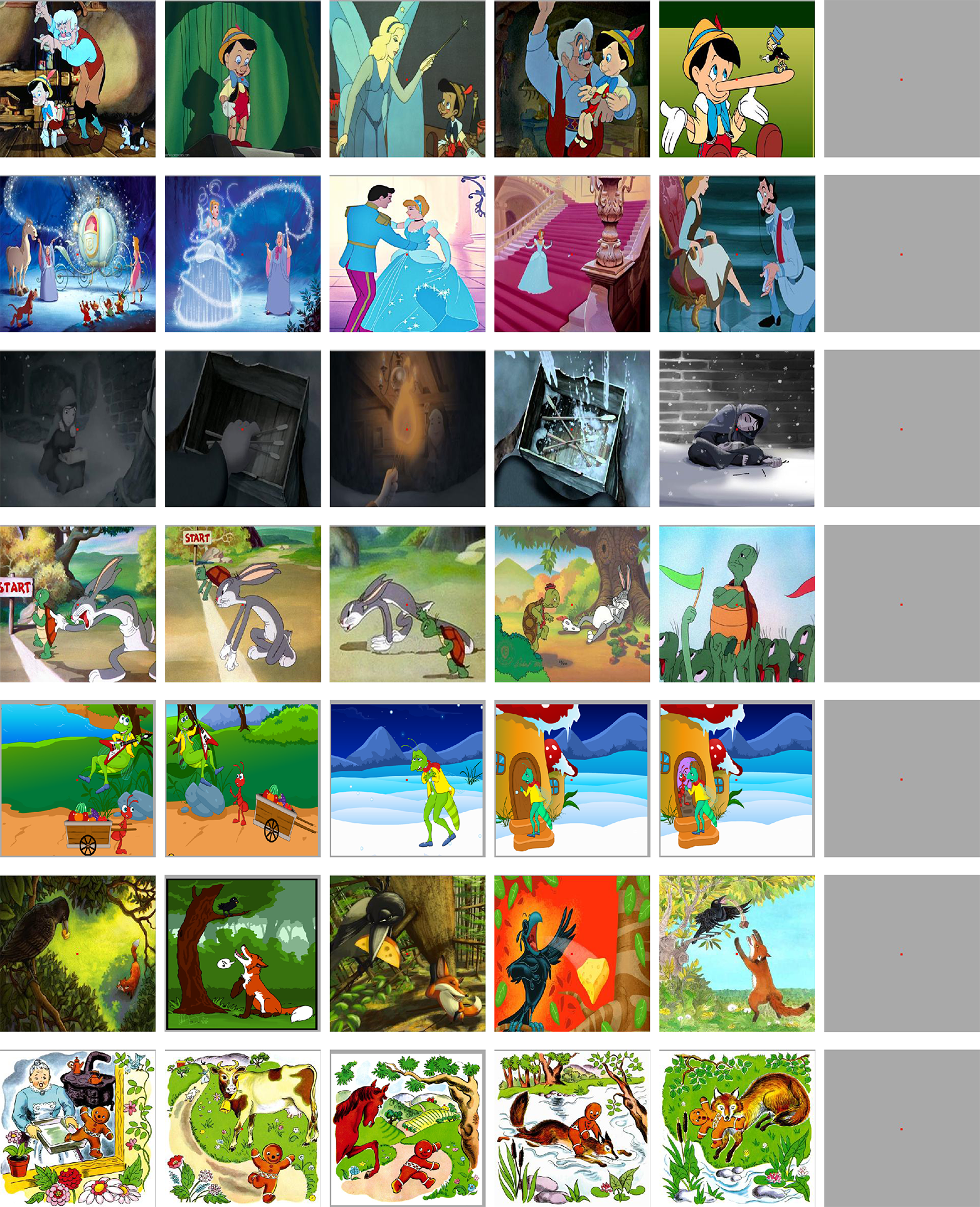

Supplement: Supplementary file 3 [file Image_2.TIF]

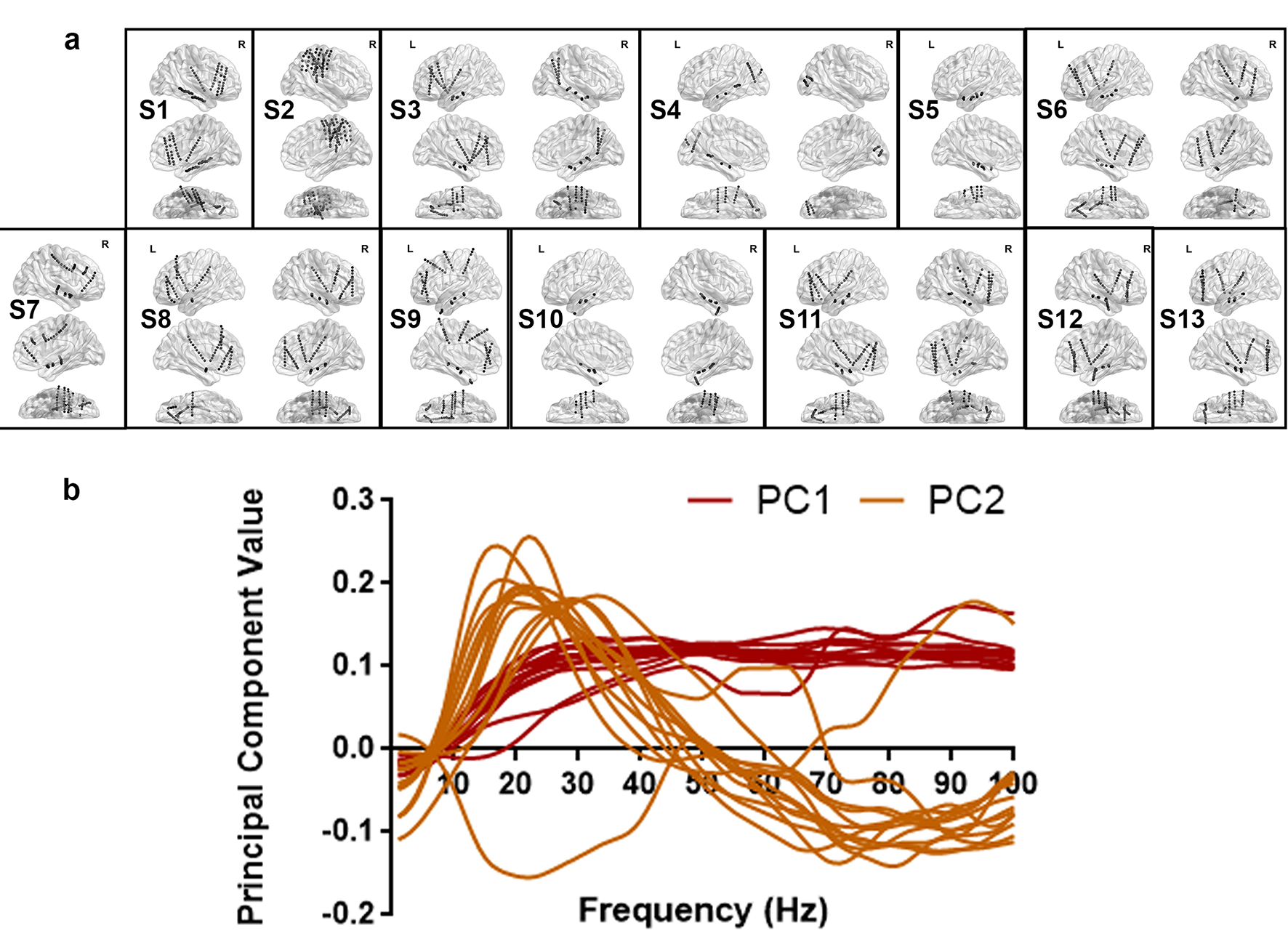

Supplement: Supplementary file 4 [file Image_3.TIF]

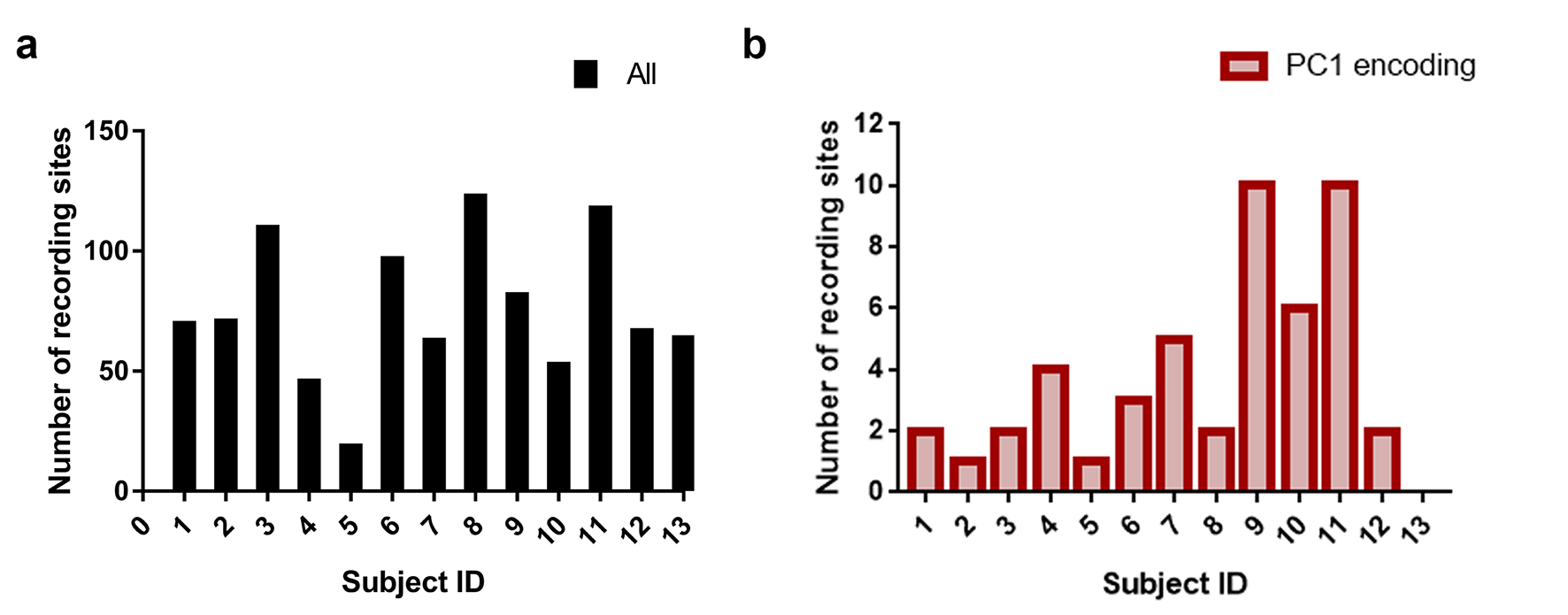

Supplement: Supplementary file 5 [file Image_4.TIF]

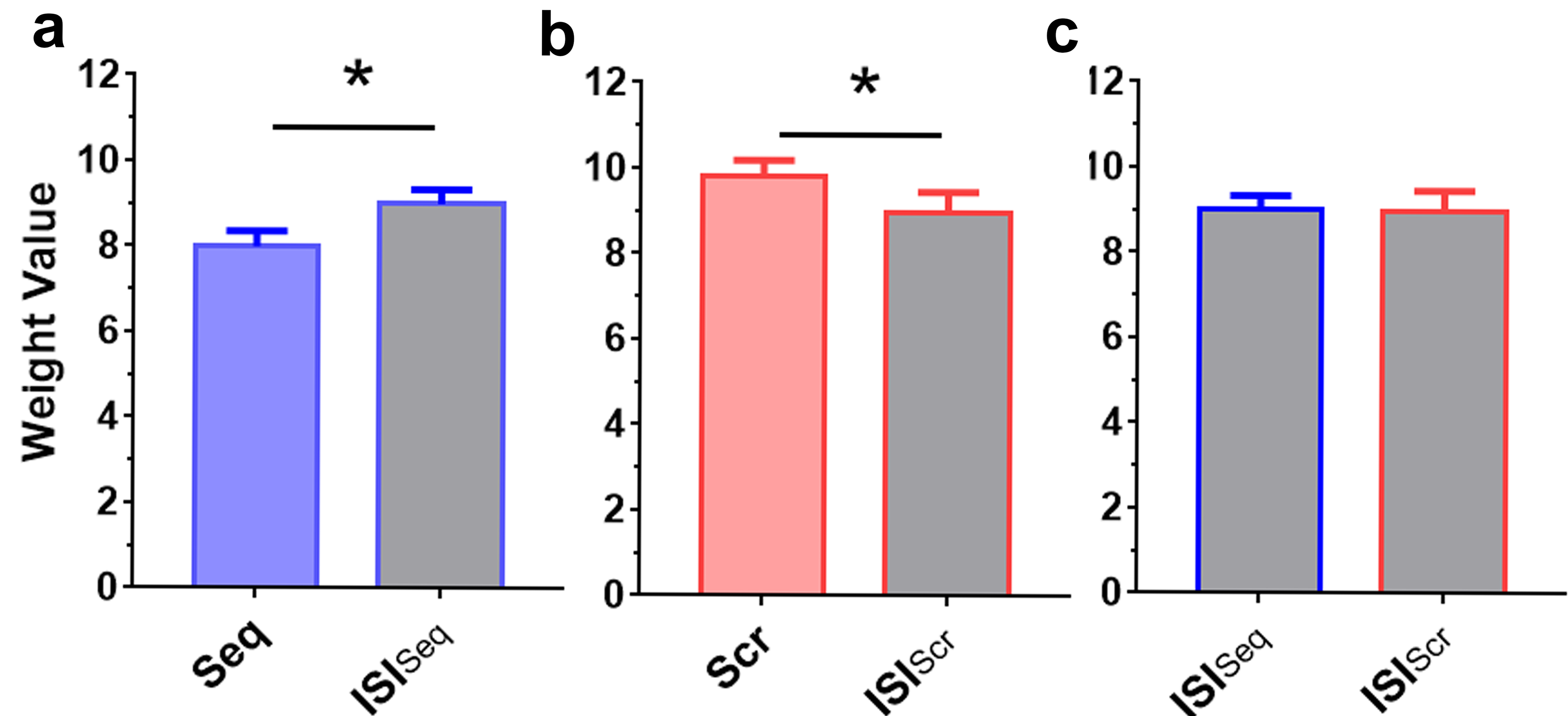

Supplement: Supplementary file 6 [file Image_5.TIF]

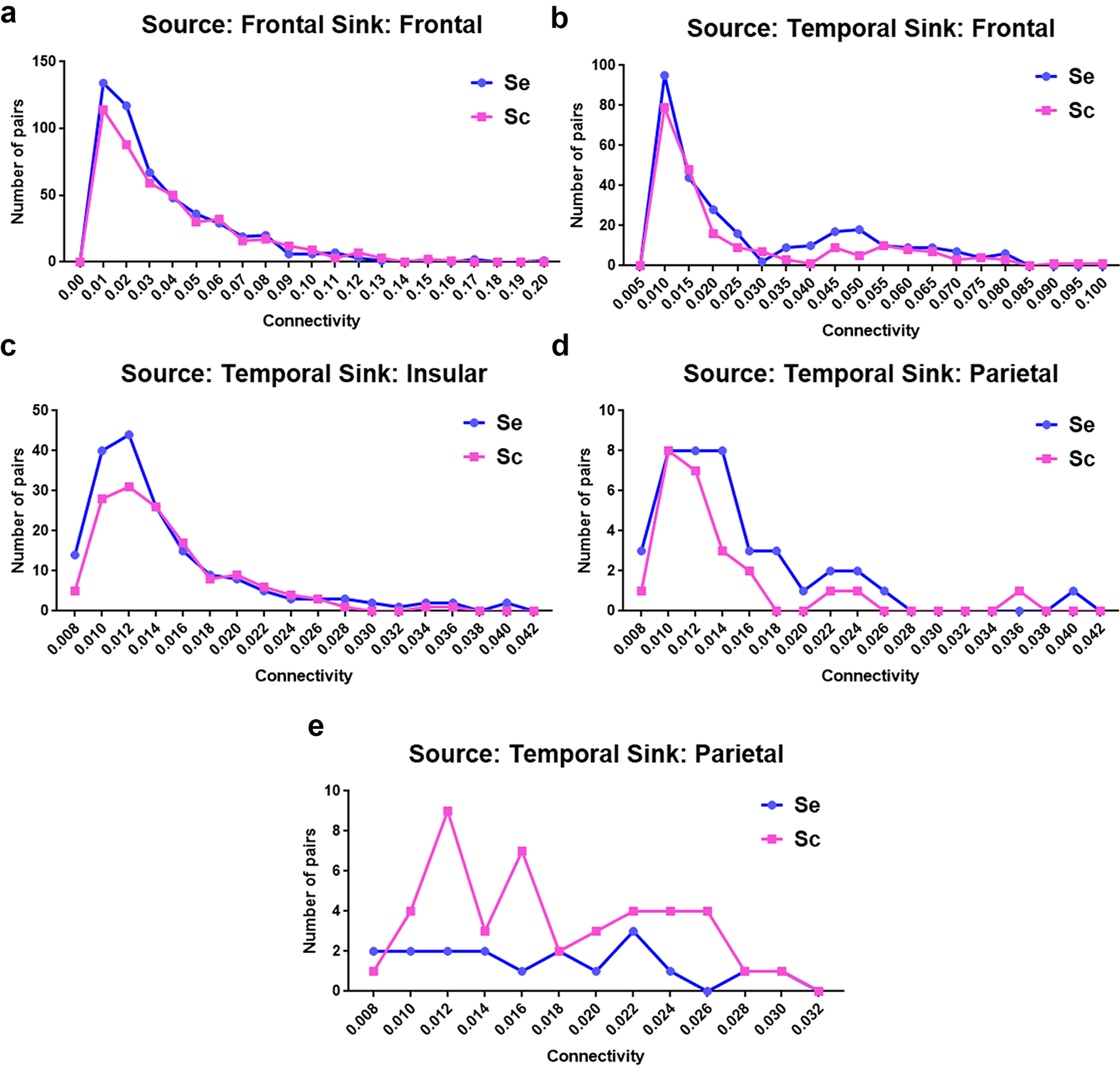

Supplement: Supplementary file 7 [file Image_6.TIF]
